# Whole-genome sequencing of multidrug-resistant *Escherichia coli* causing urinary tract infection in an immunocompromised patient: a case report

**DOI:** 10.1186/s13256-024-04663-4

**Published:** 2024-07-17

**Authors:** Mohammed Yahya Ahmed, Babbiker Mohammed Taher Gorish, Esraa Mohammed Alhaj, Mayasir Abd Elmoniem Abd Elrhim, Shimaa Saifaldeen Siddig, Hisham N. Altayb

**Affiliations:** 1 Department of Microbiology, Faculty of Medical Laboratory Science, Sudan University of Science and Technology, Khartoum, Sudan; 2 Department of Microbiology, Faculty of Medical Laboratory Science, Omdurman Islamic University, Omdurman, Sudan; 3 Department of Microbiology and Parasitology, Faculty of Medical Laboratory Science, Ibn Sina University, Khartoum, Sudan; 4 Department of Biochemistry- Faculty of Sciences, King Abdulaziz University, Jeddah, Saudi Arabia

**Keywords:** *Escherichia coli*, Whole-genome sequencing, Urinary tract infections, Antimicrobial resistance, Phylogeny tree

## Abstract

**Background:**

*Escherichia coli is* a major human pathogen responsible for a broad range of clinical illnesses. It has been linked to endemic and epidemic nosocomial diseases caused by multidrug-resistant pathogens in Sudan as well as throughout the globe.

**Case presentation:**

A 76-year-old African woman arrived at Saad Rashwan Medical Centre complaining of backaches and discomfort during urination. Throughout the preceding 5 years, the patient had recurrent urinary tract infections. Following overnight incubation at 37 °C, *Escherichia coli* was found in her midstream urine specimen on cysteine lactose electrolyte deficient agar media. Minimum inhibitory concentration (colorimetric/turbidimetric method) was employed to test a wide range of antimicrobial drugs against this bacterial strain, and the results revealed significant multidrug resistance. QIAamp^®^ DNA Mini Kit was used to obtain DNA Template from the purified *Escherichia coli* (Qiagen, Hilden, Germany). The bacterial whole-genome sequence was done by Novogene company (Hong Kong) using Illumina HiSeq 2500 (Illumina, San Diego, CA, USA), followed by whole genome reconstructions, and identification of antibiotic-resistant genes. Phylogenetic analysis revealed that our strain was related to the *Escherichia coli* DSM30083 ( genome sequence ID: CP033092.2) from the USA. Our strain possessed the following antimicrobial-resistant genes: aminoglycoside (*kdpE, baeR, cpxA, aadA5*), nitroimidazole (*msbA*), phosphonic acid (*mdtG*), tetracycline (*emrY*), macrolide, penam, tetracycline, (*evgA, TolC, H-NS*), fluoroquinolone, cephalosporin, glycylcycline, penam, tetracycline, rifamycin, phenicol antibiotic, disinfecting agents and antiseptics (*acrB; marA*), sulfonamide (*sul1*), macrolide (*Mrx*), cephalosporin, penam (*CTX-M-15*), carbapenem, cephalosporin, and penam (*OXA-1*).

**Conclusion:**

This study found that the isolated *Escherichia coli* strain had varied antimicrobial resistance genes on the basis of whole-genome sequencing and phenotypic resistance analyses. Whole-genome sequencing is critical for control and preventative methods to battle the growing threat of antimicrobial resistance. A larger investigation is recommended for improved generalization of results.

**Supplementary Information:**

The online version contains supplementary material available at 10.1186/s13256-024-04663-4.

## Introduction

Urinary tract infections (UTIs) represent the most common infection in humans and are regarded as a serious public health issue [1, 2]. Among the bacteria responsible for UTIs, *Escherichia coli* (*E. coli*) represents the most common cause of both community and nosocomial UTIs. *Proteus* spp., *Staphylococcus saprophyticus, Klebsiella* spp., and other *Enterobacteriaceae* are also UTI-causing bacteria [2, 3]. A previous UTI, female sex, obesity, diabetes, and genetic predisposition are also risk factors for UTIs [4]. Other variables, such as a damaged immune system, urinary blockage, neurodegenerative condition, and kidney damage, can possibly lead to severe type UTIs [5, 6].

In the recent decade, cephalosporins, fosfomycin, trimethoprim, fluoroquinolones, and amoxicillin in combination with beta-lactamase inhibitors have been deemed effective antibiotics for reducing the duration of *E. coli*-causing UTI symptoms [7]. However, widespread antimicrobial use has resulted in the rise of drug-resistant bacteria [8]. Since the extended-spectrum beta-lactamase-producing *E. coli* that was collected from the UTI patient showed relatively little resistance to carbapenems (imipenem, ertapenem, meropenem, and doripenem), these antibiotics were considered to be the final choice for treating UTIs [9]. The mechanisms of carbapenem resistance in *Enterobacteriaceae* are strongly linked to carbapenemase synthesis, efflux pump upregulation, and porin reduction [10]. Furthermore, the advent of mobile genetic elements containing carbapenemase has indeed been documented frequently among *Enterobacteriaceae,*, which is a serious clinical concern [11]. Next-generation sequencing (NGS) is now commonly used in epidemiological research, the study of bacterial communities, and antibiotic resistance prediction [12].

Controlling UTIs owing to multidrug resistance can be difficult, especially with the rise of resistance to antimicrobial drugs and the transfer of bacterial resistance outside of clinics into the community, notably among nursing home residents [13].

The early identification of resistance pathways by WGS has consequences for clinical trial design. In clinical trials, greater dosages or more frequent dosing may be used to overcome resistance mechanisms if they are found to only slightly raise minimum inhibitory concentrations (MICs) in comparison with the wild type MIC distributions. Furthermore, the selection of antibiotics for innovative regimens can be influenced by the identification of cross-resistance between agents through the use of WGS. The purpose of this report is to use whole-genome sequencing (WGS) on antibiotic-resistant *E. coli* strains obtained from patients to understand their genetic heterogeneity and antibiotic resistance mechanisms.

## Case presentation

**Fig. 1:**
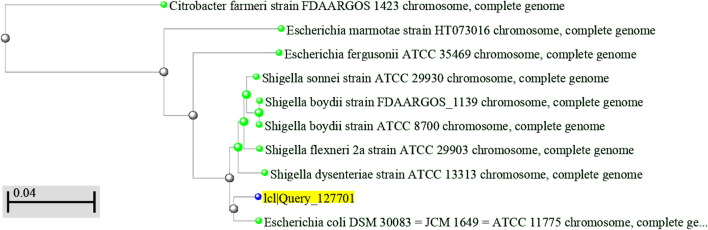
Phylogenetic trees with the highest probability. *E. coli* has traditionally been clustered by phylogroup SNP (Single Nucleotide Polymorphisms)-based phylogenetic analysis and has a good association with bacteria being microbiota or infectious by using NCBI gene phylogenetic analysis of the sequences retrieved from the GenBank as well as the sequences gained from the patient sample. Aligned were whole sequences obtained from the Gen Bank. The isolated bacteria were then assembled with those found in the gene bank using a phylogenetic tree. The results showed that our strain was close to *Escherichia coli DSM 30083* = *JCM 1649* = *ATCC 11775 chromosome, full genome sequence ID: CP033092.2* from the USA. The figure is generated by using MOLE-BLAST software, which is an experimental tool used for phylogenetic analysis

**Fig. 2:**
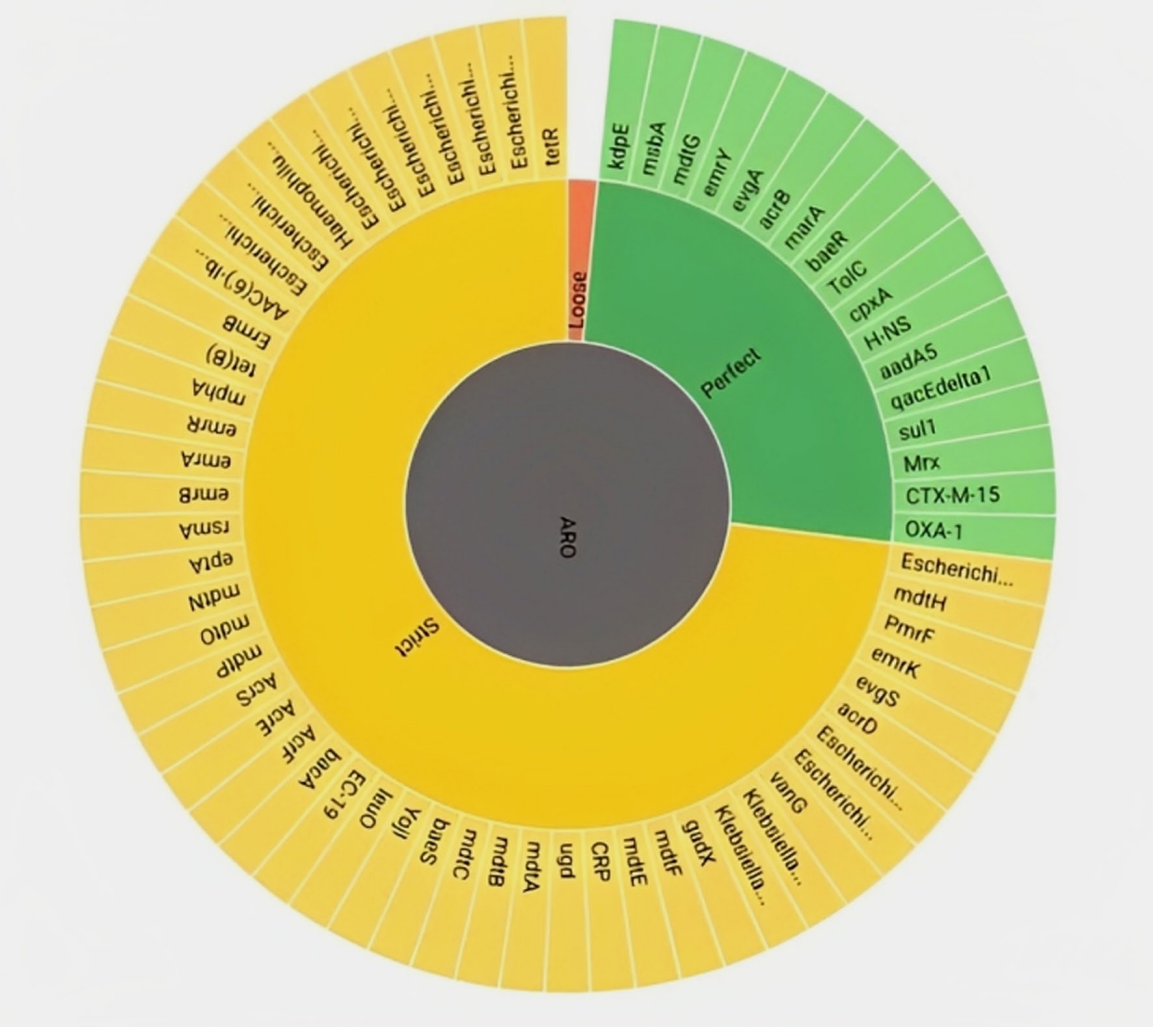
Antimicrobial resistant genes in our strain; we utilized (https://card.mcmaster.ca/analyze/rgi) for antimicrobial resistance screening. The results demonstrated that our strain possessed the following antimicrobial-resistant genes: *aminoglycoside (kdpE, baeR, cpxA, aadA5), nitroimidazole (msbA), phosphonic acid (mdtG), tetracycline (emrY), macrolide, fluoroquinolone, penam, tetracycline, (evgA, TolC, H-NS), fluoroquinolone, cephalosporin, glycylcycline, penam, tetracycline, rifamycin, phenicol antibiotic, disinfecting agents and antiseptics (acrB; marA), sulfonamide (sul1), macrolide (Mrx), cephalosporin, penam (CTX-M-15), carbapenem, cephalosporin, and penam (OXA-1*). Some antimicrobial resistant genes were present in a lower relative percentage than 100%. This figure was generated by using (https://card.mcmaster.ca/analyze/rgi) software for antimicrobial resistance screening

A 76-year-old African woman arrived at Saad Rashwan Medical Centre complaining of back pain and burning when urinating. The patient’s medical history revealed that she had diabetes and hypertension. Throughout the previous 5 years, the patient had recurrent UTIs. Additionally, she suffered from an unclear cause of anemia, and she required numerous blood transfusions. Her doctor’s most recent report stated that she was immunocompromised because of her recurrent infections. A mid-stream urine sample was inoculated using a standard loop on cysteine lactose electrolyte deficient (CLED) agar media and incubated at 37 °C overnight. Pure colonies grew the next day, and normal biochemical testing revealed that the colonies were *E. coli.* Next, the *Enterobacteriaceae* ID&AST kit was used to confirm the results (MA-120-EB). Minimum inhibitory concentration (MIC) (colorimetric/turbidimetric method) was employed to test a wide range of antimicrobial drugs against this bacterial strain, and the results revealed significant multidrug resistance. The isolated strain was resistant to ciprofloxacin, chloramphenicol, cefoxitin, ceftazidime, gentamicin, levofloxacin, piperacillin-tazobactam, cefepime, ertapenem, amoxicillin/clavulanic acid, ampicillin/sulbactam, cefuroxime, cefazolin, aztreonam, nitrofurantoin, ticarcillin/clavulanic acid, Cefoperazone/Sulbactam. Meropenem, tobramycin, minocycline, and tigecycline, however, were intermediately effective against the isolated *E. coli*. Amikacin and trimethoprim/sulfamethoxazole were effective against the isolated *E. coli*. (Table 1). QIAamp^®^ DNA Mini Kit (Qiagen, Hilden, Germany) was used to recover genomic DNA from purified *E. coli* following the manufacturer’s guidelines. The extracted DNA was measured by using the NanoDrop-800 spectrophotometer (Thermo Fisher Scientific, Wilmington, NC, USA) to determine its quality and purification. The DNA extract was completely sequenced by using the MiSeq sequencer and whole-genome pairwise sequence analysis technique, Illumina data (2 × 150 bp reads) (Illumina, San Diego, CA, USA), including whole-genome restructuring, annotation, and antibiotic-resistant gene typing. In brief, reads were trimmed with Trimmomatic to remove adaptor sequences and low-quality bases [14], and Kraken (v1.1.1) was utilized to look for possible contamination [15]. MOLE-BLAST, (https://blast.ncbi.nlm.nih.gov/Blast.cgi?PROGRAM=blastn&BLAST_SPEC=GeoBlast&PAGE_TYPE=BlastSearch) (https://pubmlst.org/bigsdb?db=pubmlst rmlst seqdef kiosk) an experimental tool that assists taxonomists in finding the nearest database neighbors of given query sequences, was used for phylogenetic analysis. It produces a phylogenetic tree by computing a multiple sequence alignment (MSA) between the query sequences and their topmost BLAST database hits. The tree’s query sequences are denoted by emphasized node labels. MOLE-BLAST may cluster input sequences from distinct genes or loci and compute an MSA and a *phylogenetic* tree for each locus sequentially. Moreover, by using a software package, the *E. coli* genome was checked for the presence of multidrug resistance and *virulence genes*. We utilized CARD (Comprehensive Antibiotic Resistance Database) (https://card.mcmaster.ca/analyze/rgi) for antimicrobial resistance screening and VFDB (Virulence Factor database) (http://www.mgc.ac.cn/cgi-bin/VFs/v5/main.cgi), a reference database for bacterial virulence factors detection for virulence gene screening. The results showed that our strain was closely related to the *Escherichia coli* DSM 30083 = JCM 1649 = ATCC 11775 chromosome, full genome sequence ID: CP033092.2, from the USA (Fig. 1). The results demonstrated that our strain possessed the following antimicrobial-resistant genes: aminoglycoside (*kdpE, baeR, cpxA, aadA5*), nitroimidazole (*msbA*), phosphonic acid (*mdtG*), tetracycline (*emrY*), macrolide, fluoroquinolone, penam, tetracycline, (*evgA, TolC, H-NS*), fluoroquinolone, cephalosporin, glycylcycline, penam, tetracycline, rifamycin, phenicol antibiotic, disinfecting agents and antiseptics (*acrB; marA*), sulfonamide (*sul1*), macrolide (*Mrx*), cephalosporin, penam (*CTX-M-15*), carbapenem, cephalosporin, and penam (*OXA-1*). Some antimicrobial-resistant genes were present in a lower relative percentage than 100% (Fig. 2) (see Additional file 1). Our findings showed that the virulence factor present in the current study strain was similar to that of *E. coli* O127:H6 str. E2348/69 (EPEC). As adhesion virulence factors, our strain employs CFA/I fimbriae, *E. coli* common pilus (ECP), *E. coli* laminin-binding fimbriae (*ELF*), EaeH, hemorrhagic *E. coli* pilus (HCP), and type I fimbriae. The current study strain, furthermore, uses the invasion of brain endothelial cells (*Ibes*) as an invasion virulence factor. The present strain’s virulence factors for heme uptake are Aerobactin siderophore, iron-regulated element, and iron/manganese transport. The current research strain employs an ACE T6SS secretion system. In addition, the present strain contains hemolysin/cytolysin A toxin and possesses capsular polysaccharide (Vibrio) as an antiphagocytic agent (see Additional file 2).

**Table 1:** MIC of the antimicrobial agents against *E. coli*

Antimicrobial agents	MIC (μg/ml)	R/I/S
Ciprofloxacin	> 4	R
Chloramphenicol	> 32	R
Meropenem	= 2	I
Cefoxitin	> 32	R
Ceftazidime	> 16	R
Gentamicin	> 16	R
Levofloxacin	> 8	R
Amikacin	< 4	S
Piperacillin/tazobactam	> 128/4	R
Cefepime	> = 16	R
Ertapenem	> 4	R
Amoxicillin/clavulanic acid	> = 32/16	R
Ampicillin/sulbactam	> 32/16	R
Cefuroxime	> = 32	R
Cefazolin	> = 8	R
Tobramycin	= 8	I
Aztreonam	> 16	R
Nitrofurantoin	> = 128	R
Ticarcillin/clavulanic acid	> = 128/2	R
Minocycline	= 8	I
Tiqecycline	= 2	I
Cefoperazone/sulbactam	> = 64/32	R
Trimethoprim/sulfamethoxazole	< = 2/38	S

*R* resistant, *I* intermediate, * S* sensitive

## Discussion

*E. coli* is a significant human infectious agent responsible for a broad range of clinical illnesses. It has been linked to endemic and epidemic nosocomial diseases caused by multidrug-resistant pathogens in Sudan and around the world [9]. Investigating the presence of antimicrobial-resistant organisms’ colonization is critical since these carriers may act as repositories for MDR microbial transmission. Furthermore, the rates are understated because of the absence of surveillance studies and effective screening procedures for individuals, as well as a concentration on patients who were admitted to healthcare institutions. WGS differs from other sequencing technologies in its ability to generate millions of reads in a single run at a low cost. The recent advancements in sequencing technology is poised to become a crucial instrument in the management of antimicrobial resistance, a serious risk to contemporary healthcare. WGS has already found many uses in this field, from the creation of new antibiotics and diagnostic tools to the monitoring and identification of the conditions that lead to the formation and maintenance of antibiotic resistance in currently prescribed medications. Since it is theoretically and economically possible to use WGS to monitor antibiotic-resistant bacteria and yield useful information for infection control, it is now necessary for laboratories to include routine genome sequencing into their surveillance plans for microbes resistant to antibiotics.

The results of this analysis revealed that our strain was close to the genome of *Escherichia coli* DSM 30083 (*genome* sequence ID: CP033092.2) from the USA, and this may reflect that bacteria recognize no borders, and worldwide trade and travel help transmit resistant germs all over the world. This adds to the intricacy of the antimicrobial resistance challenge and supports the notion that it is a worldwide problem. It makes no difference where a resistant bacterium arises. If it is likely to succeed and grows in popularity, it may travel fast to other parts of the globe in our globalized culture.

This research focused on the phenotypic resistance profiling using 17 antibiotics and the presence of resistance genes, which were verified by whole-genome sequencing. When the same drugs were tested on human *E. coli* isolates in Lusaka, Zambia and Uganda, the levels of resistance were much greater [16, 17]. The patient’s medical history revealed that she had diabetes and hypertension. Throughout the previous 5 years, the patient had recurrent UTIs. Furthermore, due to her unclear cause of anemia, she required numerous blood transfusions. The patient’s history may explain her frequent hospital visits for various reasons, including recurrent UTI antimicrobial treatment, which may have exposed her to many types of antibiotics as well as the bacteria gain resistance genes from hospitalization due to several causes and the use of many antibiotics in a short period. Furthermore, people produce and consume around 170,000 tonnes of antibiotics each year [18], making an antimicrobial resistance (AMR) emergency in humans, animals, and the environment unavoidable.

Resistance to aminoglycosides is caused primarily by the existence of aminoglycoside-modifying enzymes (*AMEs*) and their varying ability to modify aminoglycosides. In Enterobacterales, enzymes such as acetyltransferases (*AACs*), nucleotidyltransferases (*ANTs*), and phosphotransferases (*APHs*) have been described [9]. The results show that our strain carries the antimicrobial-resistant genes aminoglycoside (*kdpE, baeR, cpxA, aadA5*), which might be the cause for resistance to the antibiotics chloramphenicol and gentamicin. The strain was sensitive to amikacin and intermediately sensitive to tobramycin trimethoprim/sulfamethoxazole, which can be explained by the absence of the *aadB and aac(6′)-Ib genes*, which are specific for tobramycin and amikacin antibiotic resistance, respectively.

The isolated strain was resistant to ciprofloxacin, cefoxitin, ceftazidime, levofloxacin, piperacillin-tazobactam, cefepime, ertapenem, amoxicillin/clavulanic acid, ampicillin/sulbactam, cefuroxime, cefazolin, aztreonam, nitrofurantoin, ticarcillin/clavulanic acid, and cefoperazone/sulbactam and this may be explained by the detection of the following genes: nitroimidazole (*msbA*), phosphonic acid (*mdtG*), tetracycline (*emrY*), macrolide, fluoroquinolone, penam, tetracycline, (*evgA, TolC, H-NS*), fluoroquinolone, cephalosporin, glycylcycline, penam, tetracycline, rifamycin, phenicol antibiotic, disinfecting agents and antiseptics (*acrB; marA*), sulfonamide (*sul1*), macrolide (*Mrx*), cephalosporin, penam (*CTX-M-15*), carbapenem, cephalosporin, and penam (*OXA-1*). A similar report was found in a study conducted in Kuwait in 2022 [19]. All of the pathways identified in this study are thought to be important contributors to intrinsic and acquired multidrug resistance in Gram-negative bacteria. Our findings reveal that WGS properly predicts the mechanisms of antibiotic resistance in Enterobacterales. Because of the reduced turnaround time, use of WGS is rapidly spreading in clinical diagnostic laboratories, making genetic data more accessible and easily utilized in routine clinical settings. Advances in DNA sequence methods, plasmid conjugation, and gene cloning could considerably improve our understanding of resistant strains and their distribution and evolution.

An expanded set of virulence and AMR genes are expected to give our strains the ability to survive and thrive in the face of numerous antimicrobials in their host and environmental condition. The present strain replicons’ characterization suggests a significant amount of genetic flexibility within the plasmid-containing AMR genes. Furthermore, Sudan’s present irrational antibiotic use is predicted to enhance nosocomial and population dissemination, as well as the unrestricted development of these resistant isolates.

## Conclusion

This study found that the isolated *E. coli* strain had varied antimicrobial resistance genes on the basis of the whole genome and phenotypic resistance analyses. WGS is critical for control and preventative methods to battle the growing threat of antimicrobial resistance. A larger investigation is recommended for improved generalization of results.

### Supplementary Information

Additional file 1.

Additional file 2.

## Data Availability

All data generated or analyzed during this study are included in this published article.

## References

[1] Mazzariol A, Bazaj A, Cornaglia G (2017). Multi-drug-resistant gram-negative bacteria causing urinary tract infections: *a review*. J Chemother.

[2] Medina M, Castillo-Pino E (2019). An introduction to the epidemiology and burden of urinary tract infections. Ther Adv Urol.

[3] Alanazi MQ, Alqahtani FY, Aleanizy FS (2018). An evaluation of *E. coli* in urinary tract infection in emergency department at KAMC in Riyadh, Saudi Arabia: retrospective study. Ann Clin Microbiol Antimicrob.

[4] Foxman B (2014). Urinary tract infection syndromes: occurrence, recurrence, bacteriology, risk factors, and disease burden. Infect Dis Clin North Am.

[5] Nicolle LE, AMMI Canada Guidelines Committee (2005). Complicated urinary tract infection in adults. Can J Infectious Dis Med Microbiol.

[6] Levison ME, Kaye D (2013). Treatment of complicated urinary tract infections with an emphasis on drug-resistant gram-negative uropathogens. Current Infectious Disease Reports.

[7] Concia E, Bragantini D, Mazzaferri F (2017). Clinical evaluation of guidelines and therapeutic approaches in multi drug-resistant urinary tract infections. J Chemother.

[8] Bedenic B, Mestrovic T (2021). Mechanisms of resistance in gram-negative urinary pathogens: from country-specific molecular insights to global clinical relevance. Diagnostics.

[9] Jia P, Zhu Y, Li X, Kudinha T, Yang Y, Zhang G (2021). High prevalence of extended-spectrum beta-lactamases in *Escherichia coli* strains collected from strictly defined community-acquired urinary tract infections in adults in china: a multicenter prospective clinical microbiological and molecular study. Front Microbiol.

[10] Tilahun M, Kassa Y, Gedefie A, Ashagire M (2021). Emerging carbapenem-resistant enterobacteriaceae infection, its epidemiology and novel treatment options: a Review. Infect Drug Resist.

[11] Kopotsa K, Osei Sekyere J, Mbelle NM (2019). Plasmid evolution in carbap- enemase-producing enterobacteriaceae: a review. Ann NY Acad Sci.

[12] Price TK, Realegeno S, Mirasol R, Tsan A, Chandrasekaran S, Garner OB (2021). Validation, implementation, and clinical utility of whole genome sequence-based bacterial identification in the clinical microbiology laboratory. J Mol Diagn.

[13] Wiener J, Quinn JP, Bradford PA, Goering RV, Nathan C, Bush K (1999). Multiple antibiotic-resistant Klebsiella and *Escherichia coli* in nursing homes. JAMA.

[14] Bolger AM, Lohse M, Usadel B (2014). Trimmomatic: a flexible trimmer for Illumina sequence data. Bioinformatics.

[15] Wood DE, Salzberg SL (2014). Kraken: ultrafast metagenomic sequence classification using exact alignments. Genome Biol.

[16] Mainda G, Lupolova N, Sikakwa L, Richardson E, Bessell PR, Malama SK (2019). Whole genome sequence analysis reveals lower diversity and frequency of acquired antimicrobial resistance (AMR) genes in *E. coli* from dairy herds compared with human isolates from the same region of Central Zambia. Front Microbiol.

[17] Iramiot JS, Kajumbula H, Bazira J, de Villiers EP, Asiimwe BB (2020). Whole genome sequences of multi-drug resistant *Escherichia coli* isolated in a pastoralist community of Western Uganda: phylogenomic changes, virulence and resistant genes. PLoS ONE.

[18] Laxminarayan R, Duse A, Wattal C, Zaidi AK, Wertheim HF, Sumpradit N (2013). Antibiotic resistance-the need for global solutions. Lancet Infect Dis.

[19] Moghnia OH, Al-Sweih NA (2022). Whole genome sequence analysis of multidrug resistant *Escherichia coli* and klebsiella pneumoniae strains in Kuwait. Microorganisms.

