# Supplementary material for: Whole-genome sequencing of multidrug-resistant Escherichia coli causing urinary tract infection in an immunocompromised patient: a case report
Source: J Med Case Rep. 2024 Jul 17;18:326. doi: 10.1186/s13256-024-04663-4 (PMC11253411; doi:10.1186/s13256-024-04663-4)
Supplement: Supplementary file 1 — Additional file 1. [file 13256_2024_4663_MOESM1_ESM.docx]

| File name :Additional file 1  File format: Word document  Ttile of Data: Whole genome sequencing of multidrug-resistant *Escherichia coli* causing urinary tract infection in an immunocompromised patient: a case report.  Description of Data: Distribution of Antimicrobial resistant genes in *E. coli* strain | | | | | | | | |
| --- | --- | --- | --- | --- | --- | --- | --- | --- |
| RGI (Resistance Gene Identifier) Criteria | **ARO (Antibiotic Resistance Ontology) Term** | **Detection Criteria** | **AMR Gene Family** | **Drug Class** | **Resistance Mechanism** | **% Identity of Matching Region** | **% Length of Reference Sequence** |  |
| Perfect | kdpE | protein homolog model | kdpDE | aminoglycoside | antibiotic efflux | 100.0 | 100.00 |  |
| Perfect | msbA | protein homolog model | ATP-binding cassette (ABC) antibiotic efflux pump | nitroimidazole | antibiotic efflux | 100.0 | 100.00 |  |
| Perfect | mdtG | protein homolog model | major facilitator superfamily (MFS) antibiotic efflux pump | phosphonic acid | antibiotic efflux | 100.0 | 100.00 |  |
| Perfect | emrY | protein homolog model | major facilitator superfamily (MFS) antibiotic efflux pump | tetracycline | antibiotic efflux | 100.0 | 100.00 |  |
| Perfect | evgA | protein homolog model | major facilitator superfamily (MFS) antibiotic efflux pump, resistance-nodulation-cell division (RND) antibiotic efflux pump | macrolide fluoroquinolone, penam, tetracycline antibiotic | antibiotic efflux | 100.0 | 100.00 |  |
| Perfect | acrB | protein homolog model | resistance-nodulation-cell division (RND) antibiotic efflux pump | fluoroquinolone, cephalosporin, glycylcycline, penam, tetracycline | antibiotic efflux | 100.0 | 100.00 |  |
| Perfect | TolC | protein homolog model | ATP-binding cassette (ABC) antibiotic efflux pump, major facilitator superfamily (MFS) antibiotic efflux pump, resistance-nodulation-cell division (RND) antibiotic efflux pump | macrolide fluoroquinolone aminoglycoside carbapenem, cephalosporin, glycylcycline, cephamycin, penam, tetracycline peptide aminocoumarin rifamycin phenicol penem, disinfecting agents and antiseptics | antibiotic efflux | 100.0 | 99.60 |  |
| Perfect | cpxA | protein homolog model | resistance-nodulation-cell division (RND) antibiotic efflux pump | aminoglycoside aminocoumarin | antibiotic efflux | 100.0 | 100.00 |  |
| Perfect | H-NS | protein homolog model | major facilitator superfamily (MFS) antibiotic efflux pump, resistance-nodulation-cell division (RND) antibiotic efflux pump | macrolide fluoroquinolone cephalosporin, cephamycin, penam, tetracycline | antibiotic efflux | 100.0 | 100.00 |  |
| Perfect | sul1 | protein homolog model | sulfonamide resistant sul | sulfonamide | antibiotic target replacement | 100.0 | 100.00 |  |
| Perfect | Mrx | protein homolog model | macrolide phosphotransferase (MPH) | macrolide | antibiotic inactivation | 100.0 | 100.00 |  |
| Perfect | CTX-M-15 | protein homolog model | CTX-M beta-lactamase | cephalosporin, penam | antibiotic inactivation | 100.0 | 100.00 |  |
| Perfect | OXA-1 | protein homolog model | OXA beta-lactamase | carbapenem, cephalosporin, penam | antibiotic inactivation | 100.0 | 100.00 |  |
| Strict | Escherichia coli mdfA | protein homolog model | major facilitator superfamily (MFS) antibiotic efflux pump | tetracycline disinfecting agents and antiseptics | antibiotic efflux | 96.83 | 100.00 |  |
| Strict | mdtH | protein homolog model | major facilitator superfamily (MFS) antibiotic efflux pump | fluoroquinolone antibiotic | antibiotic efflux | 99.5 | 100.00 |  |
| Strict | PmrF | protein homolog model | pmr phosphoethanolamine transferase | peptide antibiotic | antibiotic target alteration | 99.38 | 100.00 |  |
| Strict | emrK | protein homolog model | major facilitator superfamily (MFS) | tetracycline antibiotic | antibiotic efflux | 98.86 | 110.26 |  |
