# Supplementary material for: Whole-genome sequencing of multidrug-resistant Escherichia coli causing urinary tract infection in an immunocompromised patient: a case report
Source: J Med Case Rep. 2024 Jul 17;18:326. doi: 10.1186/s13256-024-04663-4 (PMC11253411; doi:10.1186/s13256-024-04663-4)
Supplement: Supplementary file 2 — Additional file 2. [file 13256_2024_4663_MOESM2_ESM.docx]

File name: Additional file 2.

File format: Word document.

Ttile of Data: Whole genome sequencing of multidrug-resistant *Escherichia coli* causing urinary tract infection in an immunocompromised patient: a case report.

Description of Data: Distribution of Virulence factor in the *Escherichia coli* strain.

| **VFclass** | **Virulence factors** | **Related genes** | **coli_Yah(Prediction)** | **E.coli O127:H6 str. E2348/69(EPEC)** |
| --- | --- | --- | --- | --- |
|  |  |  | **draft (draft)** | **chromosome (NC_011601)** |
| Adherence | AAF/II fimbriae | aafA | - | - |
|  |  | aafB | - | - |
|  |  | aafC | - | - |
|  |  | aafD | - | - |
|  | AAF/III fimbriae | agg3A | - | - |
|  |  | agg3B | - | - |
|  |  | agg3C | - | - |
|  |  | agg3D | - | - |
|  | Afimbrial adhesin AFA-I | afaA | - | - |
|  |  | afaB | - | - |
|  |  | afaC | - | - |
|  |  | afaD | - | - |
|  |  | afaE | - | - |
|  |  | draP | - | - |
|  | CFA/I fimbriae | cfaA | orf02620 | - |
|  |  | cfaB | orf02619 | - |
|  |  | cfaC | orf02618 | - |
|  |  | cfaD/cfaE | orf02617 | - |
|  | Curli fibers | cgsD | - | - |
|  |  | cgsE | - | - |
|  |  | cgsF | - | - |
|  |  | cgsG | - | - |
|  |  | csgA | - | - |
|  |  | csgB | - | - |
|  |  | csgC | - | - |
|  | Dispersin | aap | - | - |
|  | E. coli common pilus (ECP) | ecpA | orf01213 | E2348C_0249 |
|  |  | ecpB | orf01214 | E2348C_0248 |
|  |  | ecpC | orf01215 | E2348C_0247 |
|  |  | ecpD | orf01216 | E2348C_0246 |
|  |  | ecpE | orf01217 | E2348C_0245 |
|  |  | ecpR | orf01212 | E2348C_0250 |
|  | E.coli laminin-binding fimbriae (ELF) | elfA | orf00411 | - |
|  |  | elfC | orf00413 | - |
|  |  | elfD | orf00412 | - |
|  |  | elfG | orf00414 | - |
|  | EaeH | eaeH | orf01202 | E2348C_0261 |
|  | EtpA | etpA | - | - |
|  | F1C fimbriae | focA | - | - |
|  |  | focC | - | - |
|  |  | focD | - | - |
|  |  | focF | - | - |
|  |  | focG | - | - |
|  |  | focH | - | - |
|  |  | focI | - | - |
|  | Hemorrhagic E.coli pilus (HCP) | hcpA | orf02040 | E2348C_0111 |
|  |  | hcpB | orf02041 | E2348C_0110 |
|  |  | hcpC | orf02042 | E2348C_0109 |
|  | Intimin | eae | - | E2348C_3939 |
|  | K88 fimbriae | faeC | - | - |
|  |  | faeD | - | - |
|  |  | faeE | - | - |
|  |  | faeF | - | - |
|  |  | faeG | - | - |
|  |  | faeH | - | - |
|  |  | faeI | - | - |
|  |  | faeJ | - | - |
|  | P fimbriae | papA | - | - |
|  |  | papB | - | - |
|  |  | papC | - | - |
|  |  | papD | - | - |
|  |  | papE | - | - |
|  |  | papF | - | - |
|  |  | papG | - | - |
|  |  | papH | - | - |
|  |  | papI | - | - |
|  |  | papJ | - | - |
|  |  | papK | - | - |
|  |  | papX | - | - |
|  | Porcine attaching-effacing associated protein | paa | - | - |
|  | S fimbriae | sfaA | - | - |
|  |  | sfaB | - | - |
|  |  | sfaC | - | - |
|  |  | sfaD | - | - |
|  |  | sfaE | - | - |
|  |  | sfaF | - | - |
|  |  | sfaG | - | - |
|  |  | sfaH | - | - |
|  |  | sfaS | - | - |
|  | ToxB | toxB | - | - |
|  | Type I fimbriae | fimA | orf02860 | E2348C_4621 |
|  |  | fimB | - | E2348C_4619 |
|  |  | fimC | orf02862 | E2348C_4623 |
|  |  | fimD | orf02863; orf04005 | E2348C_4624 |
|  |  | fimE | orf02859 | E2348C_4620 |
|  |  | fimF | orf02864; orf04006 | E2348C_4625 |
|  |  | fimG | orf02865 | E2348C_4626 |
|  |  | fimH | orf02866 | E2348C_4627 |
|  |  | fimI | orf02861 | E2348C_4622 |
| Autotransporter | AIDA-I type | tibA | - | - |
|  | AIDA-I | aida | - | - |
|  | AatA | aatA | - | - |
|  | Antigen 43 | agn43 | - | - |
|  | Cah | cah | orf04916 | - |
|  | Contact-dependent inhibition CDI system | cdiA | - | - |
|  |  | cdiB | - | - |
|  | EhaA | ehaA | - | - |
|  | EhaB | ehaB | orf01133 | - |
|  | Enteroaggregative immunoglobulin repeat protein | air/eaeX | orf03298 | - |
|  | EspC | espC | - | E2348C_2915 |
|  | EspI | espI | - | - |
|  | EspP | espP | - | - |
|  | Pet | pet | - | - |
|  | Pic | pic | - | - |
|  | Sat | sat | - | - |
|  | Temperature-sensitive hemagglutinin | tsh | - | - |
|  | UpaG adhesin | upaG/ehaG | orf03224 | - |
|  | UpaH | upaH | - | - |
|  | Vacuolating autotransporter gene | vat | - | - |
| Invasion | Invasion of brain endothelial cells (Ibes) | ibeA | - | - |
|  |  | ibeB | orf04772 | E2348C_0472 |
|  |  | ibeC | orf04289 | E2348C_4267 |
|  | Tia/Hek | tia | - | - |
| Iron uptake | Aerobactin siderophore | iucA | orf04834 | - |
|  |  | iucB | orf04833 | - |
|  |  | iucC | orf04832 | - |
|  |  | iucD | orf04831 | - |
|  |  | iutA | orf04830 | - |
|  | Heme uptake | chuA | orf01562 | E2348C_3743 |
|  |  | chuS | orf01563 | E2348C_3742 |
|  |  | chuT | orf01559 | E2348C_3744 |
|  |  | chuU | orf01555 | E2348C_3748 |
|  |  | chuW | orf01558 | E2348C_3745 |
|  |  | chuX | orf01557 | E2348C_3746 |
|  |  | chuY | orf01556 | E2348C_3747 |
|  | Iron-regulated element | ireA | - | - |
|  | Iron/manganese transport | sitA | orf04840 | - |
|  |  | sitB | orf04839 | - |
|  |  | sitC | orf04838 | - |
|  |  | sitD | orf04837 | - |
|  | Salmochelin siderophore | iroB | - | - |
|  |  | iroC | - | - |
|  |  | iroD | - | - |
|  |  | iroE | - | - |
|  |  | iroN | - | - |
|  | Yersiniabactin siderophore | fyuA | orf03391 | - |
|  |  | irp1 | orf03387 | - |
|  |  | irp2 | orf03386 | - |
|  |  | ybtA | orf03385 | - |
|  |  | ybtE | orf03390 | - |
|  |  | ybtP | orf03384 | - |
|  |  | ybtQ | orf03383 | - |
|  |  | ybtS | orf03381 | - |
|  |  | ybtT | orf03389 | - |
|  |  | ybtU | orf03388 | - |
|  |  | ybtX | orf03382 | - |
| LEE-encoded TTSS effectors | EspB | espB | - | E2348C_3934 |
|  | EspF | espF | - | E2348C_3930 |
|  | EspG | espG | - | E2348C_3970 |
|  | EspH | espH | - | E2348C_3944 |
|  | Mitochondria-associated protein Map | map | - | E2348C_3942 |
|  | SepZ/EspZ | sepZ | - | E2348C_3951 |
|  | Tir | tir | - | E2348C_3941 |
| Non-LEE encoded TTSS effectors | Cell-cycle-inhibitory factor Cif | cif | - | E2348C_0719* |
|  | EspFu/TccP (Tir cytoskeleton coupling protein) | espFu/tccP | - | - |
|  | EspG2 (EPEC EspC island) | espG2 | - | E2348C_2916 |
|  | EspJ | espJ | - | E2348C_0723 |
|  | EspK | espK | - | - |
|  | EspL1 | espL1 | orf01428 | - |
|  | EspL2 | espL2 | - | E2348C_3230 |
|  | EspL4 | espL4 | - | - |
|  | EspM1 | espM1 | - | - |
|  | EspM2 | espM2 | - | - |
|  | EspN | espN | - | - |
|  | EspO1-1 | espO1-1 | - | - |
|  | EspO1-2 | espO1-2 | - | - |
|  | EspR1 | espR1 | orf04044 | - |
|  | EspR3 | espR3 | - | - |
|  | EspR4 | espR4 | orf03342 | - |
|  | EspV | espV | - | - |
|  | EspW | espW | - | - |
|  | EspX1 | espX1 | orf02131 | - |
|  | EspX2 | espX2 | - | - |
|  | EspX4 | espX4 | orf04198 | - |
|  | EspX5 | espX5 | - | - |
|  | EspX6 | espX6 | - | - |
|  | EspX7 | espX7 | - | - |
|  | EspY1 | espY1 | orf02092 | - |
|  | EspY2 | espY2 | orf02079 | - |
|  | EspY3 | espY3 | - | - |
|  | EspY4 | espY4 | orf03980 | - |
|  | EspY5 | espY5 | - | - |
|  | LifA/Efa1 | lifA/efa1 | - | E2348C_3234 |
|  | NleA | nleA | - | E2348C_1442 |
|  | NleB1 | nleB1 | - | E2348C_3231 |
|  | NleB2-1 | nleB2-1 | - | E2348C_1041 |
|  | NleB2-2 | nleB2-2 | - | - |
|  | NleC | nleC | - | E2348C_1042 |
|  | NleD | nleD | - | E2348C_1044 |
|  | NleE-1 | nleE-1 | - | E2348C_3232 |
|  | NleE-2 | nleE-2 | - | E2348C_1080 |
|  | NleF | nleF | - | E2348C_1445 |
|  | NleG-1 | nleG-1 | - | - |
|  | NleG-2 | nleG-2 | - | - |
|  | NleG-3 | nleG-3 | - | - |
|  | NleG2-2 | nleG2-2 | - | - |
|  | NleG2-3 | nleG2-3 | - | - |
|  | NleG2-4 | nleG2-4 | - | E2348C_1040 |
|  | NleG5-1 | nleG5-1 | - | - |
|  | NleG5-2 | nleG5-2 | - | - |
|  | NleG6-1 | nleG6-1 | - | - |
|  | NleG6-2 | nleG6-2 | - | - |
|  | NleG6-3 | nleG6-3 | - | - |
|  | NleG7 | nleG7 | - | - |
|  | NleG8-2 | nleG8-2 | - | - |
|  | NleH1-1 | nleH1-1 | - | E2348C_0718 |
|  | NleH1-2 | nleH1-2 | - | E2348C_1444 |
|  | TccP2 | tccP2 | - | - |
| Regulation | AggR | aggR | - | - |
| Secretion system | AAI/SCI-II T6SS | aaiA | - | - |
|  |  | aaiB | - | - |
|  |  | aaiC/hcp | - | - |
|  |  | aaiD | - | - |
|  |  | aaiE | - | - |
|  |  | aaiF | - | - |
|  |  | aaiH | - | - |
|  |  | aaiI | - | - |
|  |  | aaiJ | - | - |
|  |  | aaiK | - | - |
|  |  | aaiL | - | - |
|  |  | aaiM | - | - |
|  |  | aaiN | - | - |
|  |  | clpV/aaiP | - | - |
|  |  | icmF/aaiO | - | - |
|  |  | vgrG | - | - |
|  | ABC transporter for dispersin | aatA | - | - |
|  |  | aatB | - | - |
|  |  | aatC | - | - |
|  |  | aatD | - | - |
|  |  | aatP | - | - |
|  |  | aec11 | - | - |
|  |  | aec14 | - | - |
|  |  | aec15 | orf02338; orf04776 | - |
|  |  | aec16 | orf04548 | - |
|  |  | aec17 | orf04545 | - |
|  |  | aec18 | orf04543 | - |
|  |  | aec19 | orf04542 | - |
|  |  | aec22 | orf04540 | - |
|  |  | aec23 | orf04539 | - |
|  |  | aec24 | orf04538 | - |
|  |  | aec25 | orf04537 | - |
|  |  | aec26 | orf04536 | - |
|  |  | aec27/clpV | orf04535 | - |
|  |  | aec28 | orf04534 | - |
|  |  | aec29 | orf04533 | - |
|  |  | aec30 | orf04532 | - |
|  |  | aec31 | orf04531 | - |
|  |  | aec32 | orf04530 | - |
|  |  | aec7 | - | - |
|  |  | aec8 | - | - |
|  |  | cesD2 | - | E2348C_3933 |
|  |  | cesD | - | E2348C_3956 |
|  |  | cesF | - | E2348C_3943 |
|  |  | cesT | - | E2348C_3940 |
|  |  | escC | - | E2348C_3955 |
|  |  | escD | - | E2348C_3938 |
|  |  | escF | - | E2348C_3932 |
|  |  | escI | - | E2348C_3952 |
|  |  | escJ | - | E2348C_3953 |
|  |  | escK | - | E2348C_3965 |
|  |  | escL | - | E2348C_3964 |
|  |  | escN | - | E2348C_3948 |
|  |  | escO | - | E2348C_3947 |
|  |  | escP | - | E2348C_3946 |
|  |  | escR | - | E2348C_3963 |
|  |  | escS | - | E2348C_3962 |
|  |  | escT | - | E2348C_3961 |
|  |  | escU | - | E2348C_3960 |
|  |  | escV | - | E2348C_3949 |
|  |  | espA | - | E2348C_3936 |
|  |  | espB | - | E2348C_3934 |
|  |  | espD | - | E2348C_3935 |
|  |  | etgA | - | E2348C_3959 |
|  |  | glrA | - | E2348C_3957 |
|  |  | glrR | - | E2348C_3958 |
|  |  | ler | - | E2348C_3968 |
|  |  | sepD | - | E2348C_3954 |
|  |  | sepL | - | E2348C_3937 |
|  |  | sepQ | - | E2348C_3945 |
|  | SCI-I T6SS | Undetermined | - | - |
|  |  | Undetermined | - | - |
|  |  | Undetermined | - | - |
|  |  | Undetermined | - | - |
|  |  | Undetermined | - | - |
|  |  | Undetermined | - | - |
|  |  | Undetermined | - | - |
|  |  | Undetermined | - | - |
|  |  | Undetermined | - | - |
|  |  | Undetermined | - | - |
|  |  | Undetermined | - | - |
|  |  | Undetermined | - | - |
|  |  | Undetermined | - | - |
| Toxin | Alpha-hemolysin | hlyA | - | - |
|  |  | hlyB | - | - |
|  |  | hlyC | - | - |
|  |  | hlyD | - | - |
|  | Colicin-like Usp | usp | - | - |
|  | Cytolethal distending toxin | cdtA | - | - |
|  |  | cdtB | - | - |
|  |  | cdtC | - | - |
|  | Cytotoxic necrotizing factor 1 | cnf1 | - | - |
|  | Enterotoxin 1 | set1A | - | - |
|  |  | set1B | - | - |
|  | Enterotoxin SenB/TieB | senB | - | - |
|  | Heat-labile enterotoxin | eltA | - | - |
|  |  | eltB | - | - |
|  | Heat-stable enterotoxin 1 (EAST1) | astA | - | - |
|  | Hemolysin/cytolysin A | hlyE/clyA | orf00624 | - |
|  | Shiga-like toxin | stx1A | - | - |
|  |  | stx1B | - | - |
|  |  | stx2A | - | - |
|  |  | stx2B | - | - |
| Antiphagocytosis | Capsular polysaccharide(Vibrio) | wbjD/wecB | orf01762 | - |
